# Effectiveness of a community-delivered pneumatic machine resistance training programme (Gym Tonic) for older adults at neighbourhood senior centres – a randomized controlled trial

**DOI:** 10.1186/s11556-021-00273-x

**Published:** 2021-10-07

**Authors:** Shuen Yee Lee, Alycia Goh, Ken Tan, Pei Ling Choo, Peck Hoon Ong, Wai Pong Wong, Shiou-Liang Wee

**Affiliations:** 1 Health and Social Sciences Cluster, Singapore Institute of Technology, Singapore, Singapore; 2 PulseSync Pte Ltd, Singapore, Singapore; 3 School of Health and Life Sciences, Glasgow Caledonian University, Glasgow, UK; 4 Geriatric Education and Research Institute, Singapore, Singapore

**Keywords:** Technology, Program evaluation, Community-dwelling, Physical function, Community exercise program, Muscle strength

## Abstract

**Background:**

Resistance training with pneumatic machines attenuates the age-associated loss in muscle strength and function in older adults. However, effectiveness of scaled-up pneumatic machine resistance training in the community is not known. We evaluated the effectiveness of a multi-site community-delivered 12-week pneumatic machine resistance programme (Gym Tonic (GT)) on muscle strength and physical function in older adults.

**Methods:**

Three hundred eighteen community-dwelling older adults aged ≥65 years were randomized into 12-week (twice/week) coach-supervised-community-based-GT-programme(*n* = 168) and wait-list control groups(*n* = 150). After 12 weeks, the intervention group continued with GT-training and the control group received supervised-GT-programme for further 12 weeks (partial-crossover-design). Fried frailty score, lower-extremity muscle strength and physical function (i.e., fast and habitual gait-speed, balance, repeated-chair-sit-to-stand, short physical performance battery (SPPB)) were determined at baseline, 12 and 24 weeks. Analysis adopted a modified-intention-to-treat-approach.

**Results:**

After 12 weeks, lower-extremity muscle strength improved by 11–26%(all *p <* 0.05) and fast gait-speed improved by 7%(*p* = 0.008) in GT-intervention group(*n* = 132) than controls(*n* = 118), regardless of frailty status. Other physical function performance did not differ between control and intervention groups after 12 weeks (all *p* > 0.05). Frailty score improved by 0.5 in the intervention but not control group(*p* = 0.004). Within the intervention group, lower-extremity muscle strength and physical function outcomes improved at 24 weeks compared with baseline (all *p* < 0.001). Within controls, lower-extremity muscle strength, SPPB, repeated-chair-sit-to-stand and fast gait-speed improved post-GT (24-week) compared to both pre-GT (12-week) and baseline. Programme adherence was high in intervention [0–12-weeks,90%(SD,13%); 12–24-weeks,89%(SD,17%)] and control [12–24-weeks,90%(SD,19%)] groups.

**Conclusion:**

Community-delivered GT resistance training programme with pneumatic machines has high adherence, improves muscle strength and fast gait-speed, and can be effectively implemented at scale for older adults. Future studies could examine if including other multi-modal function-specific training to complement GT can achieve better physical/functional performance in power, balance and endurance tasks.

**Trial registration:**

ClinicalTrials.gov, NCT04661618, Registered 10 December 2020 - Retrospectively registered.

**Supplementary Information:**

The online version contains supplementary material available at 10.1186/s11556-021-00273-x.

## Background

Ageing is associated with progressive loss in muscle mass and strength, especially in weight-bearing lower limb muscle groups, resulting in a decline in physical function [1, 2]. The gradual loss in muscle strength and function with age result in adverse health outcomes including frailty, increased risk of falls, lower quality of life, increased dependency, hospitalization and mortality [3, 4]. While multiple factors such as malnutrition, obesity and hormonal changes contribute to the age-associated decline in physical function, low level of physical activity (PA) is a key factor that mediates the reduction in muscle strength and function with age [5].

Despite the well acknowledged benefits of exercise on physical function and health, exercise participation in older adults remains low in many countries, including Singapore [6, 7]. Plausible factors contributing to low exercise participation rates include low accessibility of facilities, safety concerns and lack of adequate guidance with certain training modality such as resistance training [6–8]. To promote exercise participation, it is pertinent for PA research to move beyond clinical research trials to evaluate effectiveness of large-scale community-delivered programmes.

Resistance exercise, which consists of short-duration activity at high intensities of a few repetitions, is a commonly recommended exercise modality to increase muscle mass, strength, reduce frailty, maintain physical function and mobility in older adults [9, 10]. Pneumatic machines have been promoted for resistance training in older adults, as the use of compressed air to confer resistance results in reduced stress on joints, lower risk of injury and increased safety [11]. Furthermore, pneumatic machines allow older adults to scale the weight increments in smaller intervals (e.g., 100 g) than conventional plate-loaded machines, to better cater to needs of physically frail older adults. However, community-dwelling older adults have limited access to these machines, as these machines are often limited to more exclusive facilities (such as hospitals) due to its high costs and need for maintenance and support equipment, including compressor and pneumatic lines.

Compared with pneumatic machines, an acute exercise bout with conventional plate-loaded machines was of lower intensity and resulted in increased fatigue, suggesting that resistance training with pneumatic machines may be more appropriate for improving muscle strength and function in community-dwelling older adults [12, 13]. Gym Tonic (GT) programme has been implemented at nursing homes and community senior centres, in various locations around Singapore. The charity Lien Foundation initiated and scaled-up GT-programme and appointed a local aged care/health technology provider, PulseSync, to implement the programme and train coaches on the equipment, software, exercise and assessment protocols. The coaches were either exercise trainers, physiotherapists or therapy assistants. Through structured and supervised training, GT is designed to maintain physical strength and extend functional life years in older adults. GT uses pneumatic machines with standardized assessment protocols and an automated system with radio-frequency identification technology, to monitor training progress of participants and adjust the exercise load accordingly [14].

To our knowledge, no study has evaluated the effectiveness of a large multi-site community-delivered pneumatic machine resistance programme. This study evaluates the effects of the 12-week coach-led GT-training-programme implemented at three neighbourhood senior centers on muscle strength and physical function of older adults.

## Methods

### Study design

**Fig. 1:**
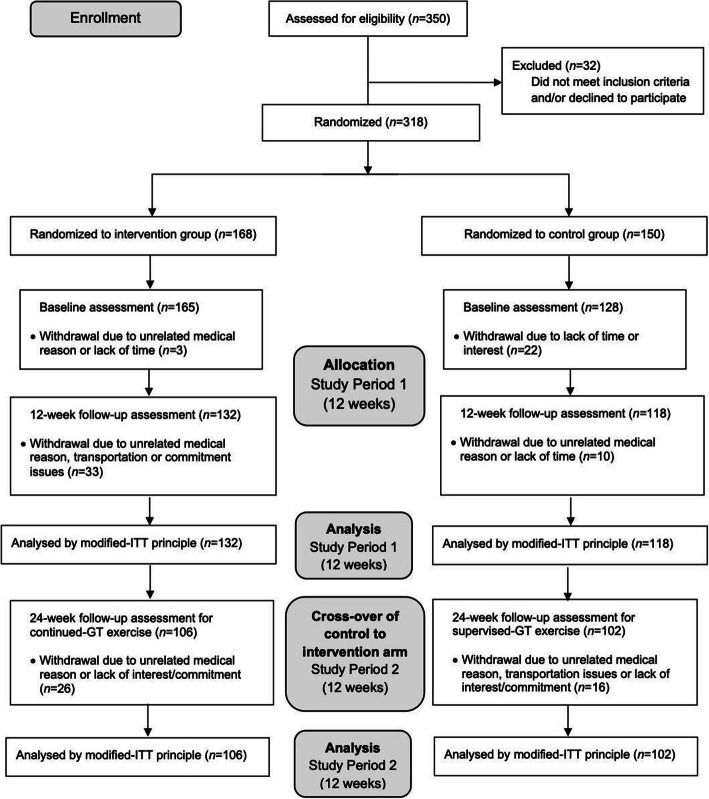
CONSORT diagram of the randomized controlled trial with crossover of control group to intervention arm after 12 weeks. GT = Gym Tonic programme, ITT = intention-to-treat

PulseSync adopted a randomized controlled design to implement the 12-week coach-led GT intervention to three community senior centers. After 12 weeks, the wait-list control group received the coach-led GT-programme for a subsequent 12 weeks, in a partial crossover design. The original intervention group had access to GT facility for a further 12 weeks (Fig. 1). Study outcomes were measured at baseline, 12 weeks and 24 weeks. All study visits were conducted across three senior centers at different residential estates in Singapore between Oct 2018 to Jul 2019 (ClincalTrials.gov Identifier: NCT04661618, retrospectively registered). All participants gave written informed consent. Researchers at Singapore Institute of Technology conducted retrospective analyses and evaluation of the study data collected by PulseSync. Ethics approval was obtained from the institutional review board of Singapore Institute of Technology (IRB-2020178).

### Participants

Community-dwelling adults aged 65 and above were recruited by Lien Foundation, PulseSync and the respective senior centres through publicity on a local radio programme and word of mouth. Respondents who were ambulant with or without the use of walking aid, and without severe cognitive impairment (i.e., Cognitive performance scale< 3 or Mini-mental state examination> 18), heart and pulmonary conditions or conditions that were contraindicative to exercise, were screened and included in the GT-programme by coaches at the respective centres. For two of the three centres, participants were randomised 1:1 to intervention or control groups in blocks of 4 or 10 using a computer-generated randomization list, by coaches. For the last centre, randomization was unequal (3:2) with a larger intervention group, as more respondents were unwilling to wait for 12 weeks prior to exercise intervention. Group allocation was concealed until participants were enrolled. Neither participants nor assessors were blinded to the intervention allocation.

### Intervention

The exercise intervention consisted of twice-weekly coach-supervised progressive resistance training for 12 weeks, with a maximum coach-to-participant ratio of 1:6. The exercises included chest press, lat pulldown, abdomen, back extension, leg press, leg extension, leg curl, hip abduction and hip adduction, which target major upper and lower body muscle groups. Within the 12-week training period, participants underwent 2-week training at 15 RM for 2 sets of 15 repetitions (~ 60% 1RM). For the subsequent 5 weeks, each training session involved a workload at 10 RM for 2 sets of 10 repetitions (~ 70% 1RM). During the last 5 weeks, participants performed a workload of 8 RM for 2 sets of 8 repetitions (~ 80% 1RM) [9]. The intensity, volume and progression of training were based on the current recommended resistance training guidelines for older adults [9, 15]. A minimum of 15 s recovery was provided between sets. The load increment was determined by coaches, ranging from 0.1–1 kg when participants could perform 2 additional repetitions per set.

After the initial 12-week wait, the control group completed the same 12-week GT-programme. Participants in the original intervention group continued a further 12 weeks of GT training, involving 3 sets of 10 repetitions at 10 RM twice-weekly. All exercises were performed using pneumatic machines (HUR, Kokkola, Finland).

### Outcomes

#### Anthropometric and cardiovascular

Anthropometric measures included body weight, height, body mass index (BMI) and waist circumference. Blood pressure and heart rate were measured with an automated device (Omron Corporation, Kyoto, Japan). All measures were performed by the senior centre coaches.

#### Muscle strength

Handgrip strength was measured with participants seated with arms unsupported, elbow joint at 90 degrees and wrist in a neutral position while holding the hand dynamometer (Jamar Plus+, Patterson Medical, Cedarburg, WI). The highest reading of three trials per arm with 30s rest intervals was reported. Lower body muscle strength was measured using 1 RM for left and right knee flexion and extension, hip abduction and hip adduction in both limbs (HUR, Kokkola, Finland), with the highest reading of three trials reported. All muscle strength assessments were also conducted by respective coaches at each senior centres.

#### Physical function

Physical function was assessed by the respective trained coaches at each senior centre, using the short physical performance battery (SPPB) and fast gait speed (GS). SPPB assesses lower limb function, including balance, strength and mobility. SPPB consisted of 3 subtests (balance, habitual GS and sit-to-stand time) [16]. The balance subtest comprised 3 parts each with progressive difficulty, including unaided feet-together stand, semi-tandem stand and full-tandem stand. Participants were timed until they moved or maximum of 10s. The repeated-chair sit-to-stand assessment involved a pre-test to determine if participants could stand up from the chair with arms across chest. Participants were timed while performing five chair stands as quickly as possible. Habitual GS was calculated by timed walk of 4 m at usual pace. To account for acceleration and deceleration, markers were provided 1.5 m before and after the measured distance. Each of the 3 subtests was scored from 0 to 4 and the total score was the sum of 3 subtests, ranging from 0 to 12. Higher SPPB scores indicated better physical function [16]. Fast GS was similarly assessed by determining participants timed walk over 4 m, as fast and comfortably able, with a moving start.

#### Frailty status

Frailty status was assessed using Fried’s Frailty phenotype, which defines frailty based on presence of five components: weakness, unintentional weight loss, slowness, exhaustion and low PA [17]. Weakness was determined using the Asian Working Group for Sarcopenia hand grip strength criteria of < 26 kg for men and < 18 kg for women [18]. Unintentional weight loss was defined as weight loss of ≥4.5 kg in 6 months or BMI ≤18.5 kg/m^2^. Slowness was identified as habitual walking speed (4 m) stratified by sex and height [17]. Exhaustion was determined through a self-reported 2-item questionnaire adapted from the Centre for Epidemiologic Studies Depression [19]. Participants were classified as low PA if they engaged in less than once per week of at least 30mins of self-reported moderate-to-vigorous-intensity PA [17, 20]. Participants were classified into frailty status based on presence of number of components: robust (0), pre-frail (1, 2) and frail (3–5).

### Statistical analysis

All statistical analyses were performed using R version 3.6.2 (R Foundation for statistical computing, Vienna, Austria). A sample size of 200 (100 per group) was needed for the trial to have 80% power to detect a two-sided hypothesis test at an α level of 0.05 (effect size of 0.2) (G*Power, version 3.1, Germany). After allowing for a 30% drop-out rate, the planned sample size was 150 participants per group. All participants with completed outcome measures at baseline and 12 weeks were included for analysis according to their randomized group allocation (modified intention-to-treat analysis), and maximum likelihood estimation was used to analyse missing data. Numerical variables are presented as mean (standard deviation, SD) in text and figures unless otherwise stated. Independent samples t-test was used to compare differences in participant characteristics between control and intervention groups. Between-group differences for physical function, muscle strength, anthropometric and cardiovascular outcomes measured from baseline to 12 weeks were analysed using mixed-effect model with Group, Time, and their interactions as fixed effects and subject as random effect. For between-group effects of frailty status on physical function and muscle strength across 12 weeks, a similar mixed-effect model with Group, Time, Frailty status, and their interactions as fixed effects and subject as random effect was used. All mixed-effect models were adjusted for age, sex and moderate-to-vigorous-PA levels at baseline. Bonferroni adjustment was used for comparison of timepoints (baseline–12 weeks) between groups. Within-group differences for pre- to post-GT in the entire cohort were analysed using the paired t-test. One-way ANOVA with Bonferroni adjustment was used to analyse within-group differences in outcome measures from baseline to 12 and 24 weeks. A value of *p* < 0.05 was considered statistically significant.

## Results

### Participant characteristics

Three hundred eighteen participants were recruited and randomized into intervention (*n* = 168) and wait-list control (*n* = 150) groups. Prior to baseline measurement, 3(2%) and 22(15%) participants withdrew from the intervention and control groups respectively, due to unrelated medical conditions, lack of time or interest (Fig. 1). A total of 165 (75.8% women) and 128 (72.7% women) participants in the intervention and control groups completed baseline assessment respectively. From baseline to 12 weeks, 33(20%) participants from the intervention group and 10(8%) participants from the control group withdrew, due to unrelated medical conditions, transportation or commitment issues. After 12 weeks, 132 (77.3% women) and 118 (72.9% women) participants started and completed the GT intervention and control phase, respectively, and had outcome data for analysis (Fig. 1). Between week 12–24, 102 participants (72.5% women) from the original control group completed the GT-programme, allowing for a total of 234 participants in the longitudinal analysis. From week 12–24, 106 participants (77.4% women) from the original intervention group continued and completed the GT-programme (Fig. 1).

Among participants who completed post-testing, mean adherence to 12-week GT-programme was 90%(SD,13%) for intervention (baseline–12 weeks), 89%(SD,17%) for wait-list control (12–24 weeks) groups, and 90%(SD,19%) for intervention (12–24 weeks) group.

Participant baseline characteristics are presented in Table 1, with no difference between groups, apart from PA. Intervention group engaged in ~ 0.5 more days/week of ≥30 min of moderate-to-vigorous-intensity PA at baseline (*p* = 0.02). The results presented did not differ across community senior centres; hence, data are presented with centre excluded from the model.

**Table 1:** Mean (SD) Participant characteristics at baseline in control and intervention groups

	Control	Intervention	***P*** value
*n*	128	165	
Sex, Female (n (%))	92 (74%)	125 (76%)	
Age (years)	73 (6)	73 (7)	0.836
Height (cm)	156 (8)	156 (8)	0.693
Weight (kg)	58 (12)	57 (12)	0.619
Body Mass Index (kg/m2)	23.7 (4.1)	23.4 (4.2)	0.493
Waist Circumference (cm)	86.5 (11.3)	86.8 (11.6)	0.818
Systolic BP (mmHg)	130 (16)	129 (17)	0.668
Diastolic BP (mmHg)	76 (10)	77 (10)	0.816
Heart Rate (bpm)	73 (11)	73 (11)	0.945
Mod/Vig Physical Activity (days/wk)	1.0 (1.8)	1.5 (2.1)	0.02*
Frailty Status (%)			
*Robust*	24	23	
*Pre-Frail*	56	61	
*Frail*	20	16	

*Mod* Moderate intensity, *Vig* Vigorous intensity, *p < 0.05

### Anthropometry, cardiovascular and frailty status

After 12 weeks, individuals in the intervention group had 2% decrease in waist circumference, which was not apparent in controls (Group×Time;*p* = 0.015,Table 2). Cardiovascular outcomes did not differ between groups after 12 weeks (Table 2). Frailty score improved by 0.5 points in the intervention group but not the control group after 12 weeks (Group×Time; *p* = 0.004,Table 2).

**Table 2:** Mean (SD) Study outcomes at 12 weeks in GT-intervention and control groups

	Control (***n*** = 118)			Intervention (***n*** = 132)			***P*** value
	Baseline	12-week	Change	Baseline	12-week	Change	Group× Time
**Anthropometric**							
Weight (kg)	57.9 (11.8)	57.8 (11.8)	−0.1 (1.1)	56.7 (11.8)	57.0 (11.8)	0.3 (1.4)	0.054
Body Mass Index (kg/m2)	23.6 (3.8)	23.6 (3.9)	0.0 (0.5)	23.1 (4.3)	23.2 (4.4)	0.1 (0.6)	**0.034**
Waist Circumference (cm)	86.3 (11.5)	86.4 (10.7)	0.1 (5.0)	86.1 (11.9)	84.5 (12.0)**	−1.6 (5.7)	**0.015**
**Cardiovascular**							
Systolic BP (mmHg)	130 (16)	129 (16)	−2 (15)	130 (17)	128 (13)	−1 (14)	0.842
Diastolic BP (mmHg)	76 (10)	75 (9)	−1 (8)	76 (10)	75 (9)	−2 (8)	0.275
Heart rate (bpm)	73 (11)	74 (13)	1 (9)	74 (11)	76 (11)	2 (8)	0.416
**Muscle Strength**							
Hand Grip (kg)	21.3 (6.4)	21.5 (6.3)	0.2 (2.3)	21.3 (6.9)	21.4 (6.3)	0.1 (2.8)	0.616
Knee Extensor Left (kgf)	52.7 (19.8)	56.6 (22.8)	4.0 (14.1)	54.4 (22.0)	60.9 (22.9)	6.6 (14.4)	0.148
Knee Extensor Right (kgf)	53.2 (21.8)	56.7 (21.7)*	3.6 (11.7)	53.4 (20.5)	61.6 (22.2)***	8.2 (15.9)	**0.010**
Knee Flexion Left (kgf)	27.3 (11.5)	28.9 (12.2)	1.5 (7.3)	27.2 (13.0)	32.4 (13.8)***	5.2 (8.5)	**< 0.001**
Knee Flexion Right (kgf)	26.9 (26.5)	28.2 (12.8)	1.2 (9.2)	26.5 (11.9)	33.3 (13.5)***	6.8 (9.2)	**< 0.001**
Hip Abduction (kgf)	43.8 (13.3)	44.3 (13.8)	0.5 (7.0)	44.7 (14.0)	49.4 (14.6)***	4.6 (7.0)	**< 0.001**
Hip Adduction (kgf)	47.6 (17.2)	49.4 (18.8)	1.8 (8.7)	47.9 (16.4)	54.8 (19.0)***	6.9 (9.6)	**< 0.001**
**Physical Function**							
5x Sit-to-stand (s)	13.4 (7.7)	12.9 (9.0)	−0.4 (4.1)	12.6 (5.7)	11.1 (5.3)	−1.2 (3.9)	0.105
Habitual Gait Speed (m/s)	0.93 (0.34)	1.00 (0.33)	0.06 (0.22)	0.95 (0.29)	1.04 (0.35)	0.10 (0.22)	0.267
Fast Gait Speed (m/s)	1.35 (0.51)	1.37 (0.48)	0.02 (0.30)	1.31 (0.39)	1.40 (0.48)***	0.11 (0.26)	**0.008**
Balance Score	3.46 (1.05)	3.64 (0.79)	0.17 (0.89)	3.53 (0.92)	3.54 (0.99)	0.07 (0.75)	0.252
SPPB Score	9.7 (3.0)	10.3 (2.6)	0.5 (1.6)	10.1 (2.5)	10.5 (2.7)	0.5 (1.3)	0.861
Fried Frailty Score	1.52 (1.16)	1.34 (1.08)	−0.16 (0.78)	1.40 (1.07)	0.96 (1.05)***	−0.49 (0.89)	**0.004**

*p < 0.05, **p < 0.01, ***p < 0.001 significant at 12-week compared to values at baseline within group; Bold type interface indicates significant interaction effect (Group×Time), *p* values adjusted for age, sex and physical activity levels at baseline

### Muscle strength

**Fig. 2:**
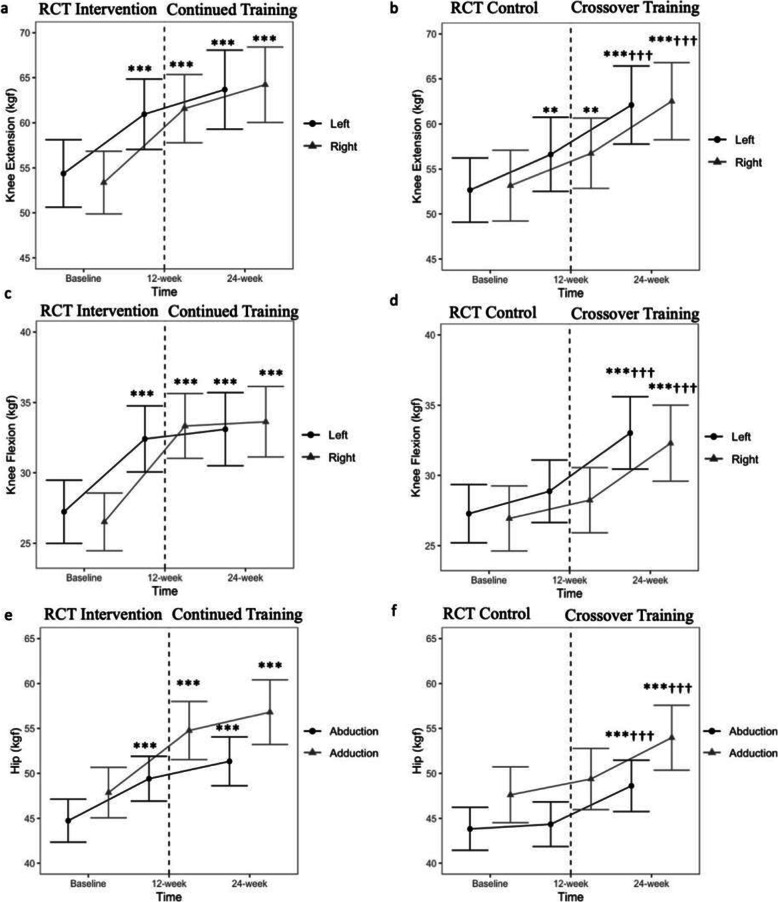
Muscle strength outcomes including knee extension (**a**, **b**) and knee flexion (**c**, **d**), and hip abduction and adduction (**e**, **f**) at baseline, 12 weeks and 24 weeks between intervention (**a**, **c**, **e**) and control (**b**, **d**, **f**) groups. For panels **a**–**d**, black circle indicates left knee and grey triangle indicates right knee, while black circle represents hip abduction and grey triangle represents hip adduction in panels e–f. Data presented are mean and 95% CI. **p < 0.01, ***p < 0.001 compared to values at baseline; †††*p* < 0.001 compared to values at 12 weeks. RCT = randomized controlled trial phase, Crossover = crossover of control group to intervention arm. Intervention = Gym Tonic resistance training programme

Hand grip and left knee extension strength did not differ between control and intervention group after 12 weeks (Group×Time;*p* > 0.05, Table 2). After 12 weeks, right knee extension increased by 15% among the intervention group (Group×Time;*p* = 0.01,Table 2). Knee flexion strength increased by 19–26%, and hip abduction and adduction strength increased by 11–14% after 12 weeks in the intervention but not control group (Group×Time; *p* < 0.001,Table 2). Left and right knee extension and flexion, hip abduction and adduction strength increased after 12- and 24-week GT-programme, compared with baseline in the intervention group (Fig. 2a, c, e), and post-GT (24-week) compared with both baseline and pre-GT (12-week) in the control group (Fig. 2b, d, f).

Among 234 participants from both groups who completed the 12-week GT-programme, knee extension and flexion, hip abduction and adduction strength increased by 10–20% (all *p* < 0.001, Fig. S1). Baseline frailty status did not affect right knee extension, left and right knee flexion, hip abduction and adduction strength between groups from baseline–12 weeks (all *p* > 0.05). In pre-frail individuals only, the increase in left knee extension strength was apparent in the intervention (19%, *p* < 0.001) but not control group (6%, *p* = 0.237) (Group×Time×Frailty; *p* = 0.013).

### Physical function

**Fig. 3:**
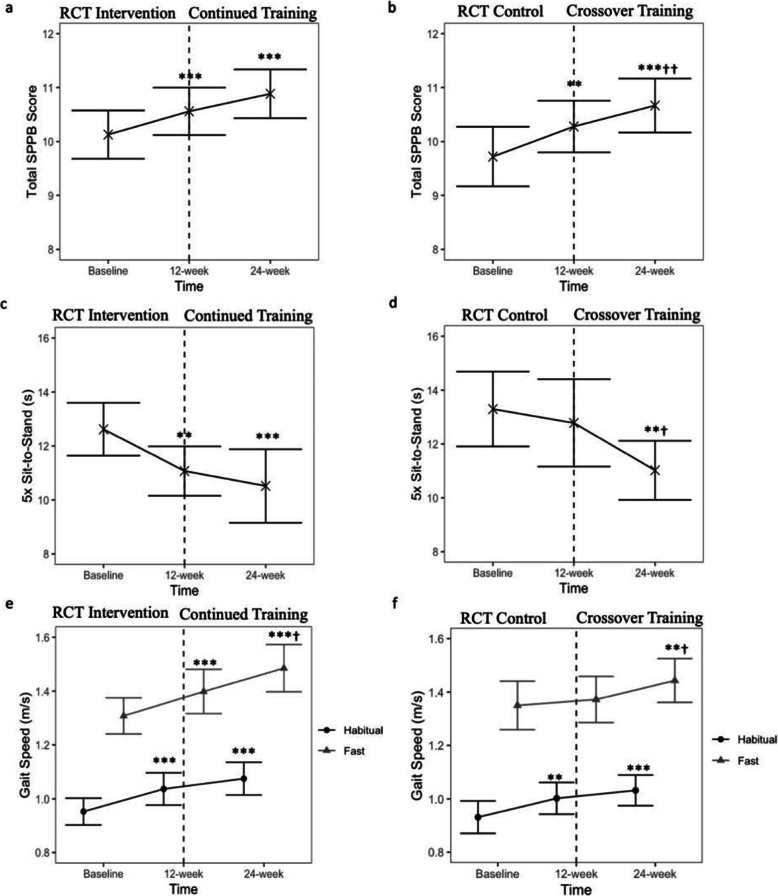
Physical function outcomes including total short physical performance battery scores (**a**, **b**), five times repeated chair sit-to-stand time (**c**, **d**), and habitual (black circle) and fast gait speed (grey triangle) (**e**, **f**) at baseline, 12 weeks and 24 weeks between intervention (**a**, **c**, **e**) and control (**b**, **d**, **f**) groups. Data presented are mean and 95% CI. **p < 0.01, ***p < 0.001 compared to values at baseline; †*p* < 0.05, ††*p* < 0.01 compared to values at 12 weeks. RCT = randomized controlled trial phase, Crossover = crossover of control group to intervention arm. Intervention = Gym Tonic resistance training programme

Repeated chair sit-to-stand time, habitual GS and SPPB total score did not differ between intervention and control groups after 12 weeks (Group×Time;all *p* > 0.05,Table 2). Within intervention group, SPPB, sit-to-stand and habitual GS improved at 12 and 24 weeks than baseline (all *p <* 0.01,Fig. 3a, c, e). Within control group, SPPB and sit-to-stand improved post-GT (24-weeks) compared with pre-GT (12-weeks) and baseline (all *p <* 0.05,Fig. 3b, d), while habitual GS improved post-GT (24-weeks) compared with baseline only (*p* < 0.001, Fig. 3f). Balance score from baseline to 12 weeks did not differ between groups (Group×Time; *p* = 0.252,Table 2). Balance score did not differ within intervention group across 24 weeks (all *p* > 0.05). Within controls, balance score improved post-GT (24-weeks) compared to baseline (*p* = 0.02).

After 12 weeks, fast GS increased by 7% in the intervention but not the control group (Group×Time; *p* = 0.008,Table 2). Within the intervention group, fast GS at 24-weeks post-GT was higher than both 12-weeks post-GT (*p* = 0.02) and baseline (*p* < 0.001) (Fig. 3e). Within the control group, fast GS was higher post-GT (24-weeks) than both pre-GT (12-weeks) (*p* = 0.037) and baseline (*p* = 0.004) (Fig. 3f).

Among all 234 participants who completed the 12-week GT-programme, SPPB and sit-to-stand improved by 3.6% and 11.7% respectively, while habitual and fast GS improved by 6.0% (all *p* < 0.001, Fig. S2a–d). Balance score did not differ after 12-week GT (*p* = 0.131, Fig. S2e). Physical function measures between groups did not differ by baseline frailty status after 12 weeks (all *p* > 0.05).

## Discussion

The present study is the first to evaluate the effectiveness of multi-site community-delivered 12-week GT-programme (pneumatic machine resistance training) among older adults. The GT-programme can be implemented in neighbourhood senior centres and is effective in improving muscle strength and fast GS regardless of frailty status. GT-programme provides structured guidance, progression and facility for resistance training in a safe environment, which promotes exercise initiation, adherence and maintenance among community-dwelling older adults.

An increase in muscle strength, especially in lower extremities, is associated with a reduction in fall risk [21], and improvement in physical function, quality of life and mental health in older adults [22]. Compared with controls, our results showed 11–26% improvement in lower-extremity muscle strength with GT intervention from baseline to 12 weeks. The increase in muscle strength was also maintained after 12-week continued-training in the intervention group. Maintaining muscle strength with age is important to reduce associated personal, social and economic burdens [23]. Our results extended the findings of previous small research studies on muscle strength improvement with pneumatic machine resistance training [24, 25], to a community programme implemented at multiple neighbourhood senior centres.

GT-programme increased fast GS on average by 0.1 m/s in the intervention group compared with controls, from baseline to 12 weeks. GS is an important predictor for fall risk, disability and survival [26–29]. In a large pooled analysis of 34,485 community-dwelling older adults, GS increment of 0.1 m/s was clinically significant, resulting in better survival [29]. Accelerated decline in fast GS predicted future disability in older adults, independent of baseline GS [27]. These results suggest that GT-programme-induced improvement in mobility may mitigate some age-associated functional decline, plausibly improving survival and disability among older adults. A systematic review of 24 studies reported that resistance training improved fast gait by a greater extent than habitual GS [30]. In contrast with our findings, a meta-analysis of 16 studies concluded that strength and combination (aerobic and other exercises) training had no effect on fast GS [31]. The disparity in findings could be due to inclusion of studies without a control group [32], and participants from tertiary-care hospitals who were frail and at risk of falls [33], which differed from our randomized controlled study of community-dwelling adults with varying frailty status. Baseline fast GS of GT-programme participants was slightly slower than reference values of community-dwelling older adults of the same age group who were independent in performing activities of daily living [34]. These results imply lower mobility at baseline among GT-programme participants, evidenced by the majority of participants with pre-frailty and frailty, which could explain the clinically significant response of fast gait to GT training. The mechanisms underlying the improvement in fast GS with GT programme may involve an increase in hip extensor and ankle plantar flexor strength with exercise through greater activation of motor units, contributing to improvement in neuromuscular and cognitive function, and in turn resulting in faster GS [35, 36].

GT-programme did not significantly affect other measures of physical function performance compared to controls. The programme utilised a single-mode resistance training with pneumatic machines, which might not be as effective as multi-modal exercise, targeted at improving specific functional performance. These findings highlight the importance of training specificity in older adults. In addition to resistance exercise, targeted training programmes for power, flexibility, balance and/or endurance, can be complementary to mitigate other age-related physical or functional decline [37–39].

Across all GT-programme participants, SPPB, habitual GS and sit-to-stand improved after 12 weeks compared to pre-training, suggesting an increase in physical function, which could improve mobility, disability and quality of life [40]. However, balance performance did not improve with GT-programme. In contrast, earlier studies observed an improvement in balance after 10–12-week resistance training among older adults aged ≥65 years [41, 42]. Unlike earlier studies which measured balance performance on a continuous scale without an upper limit, balance performance in our study might not be sensitive enough to observe improvements with GT-programme. In agreement with SPPB scores from community-dwelling older Singaporean adults [43], pre-GT balance scores in this study were also close to maximum (~ 3.5), which could plausibly decrease the potential for a greater response to the intervention in our population. Given the potential ceiling effects of SPPB and subtest scores, future studies should employ other measures of balance performance and report specific test values instead of ordinal scores.

Higher exercise participation history predicts long-term exercise adherence [44, 45]. In our study, adherence was high (~ 90%) among participants who completed the GT-programme, independent of baseline PA levels. Our findings suggest that community-delivered GT is feasible and engaging, even for participants who were physically inactive at baseline. Also, small-group, supervised and individualised nature of GT-programme could plausibly increase exercise adherence [45, 46]. Nonetheless, the drop-out rate for GT-programme across all participants was 13–20%, due to factors such as transportation or commitment, suggesting that environmental factors (e.g., accessibility and flexibility) plausibly influence exercise behaviours among older adults, especially for those with poor mobility [46]. While study participants were not charged for 12-week GT programme, community providers charge a package fee (SGD150–250), with a waitlist at popular sites, showing that the programme has been successfully scaled-up in the community. The present study is a pragmatic randomized controlled trial, which focused on the evaluation of the effectiveness and implementation of GT programme in the real-world setting. Hence, apart from muscle strength and function outcomes, evaluation of adherence and scalability potential of GT programme is also salient.

This community implementation research incorporated a wait-list control design to incentivise retention in the control group and to allow for longitudinal analysis and comparison between pre- and post-GT exercise intervention among the entire cohort. There are limitations owing to nature of implementation. The improvements in muscle strength and function in the control group from baseline to 12-weeks may likely be due to the Hawthorne effect [47], where the participant’s behaviour is modified due to awareness of being observed, or the recruitment of highly motivated participants in the control group. Furthermore, the assessments were performed by coaches who were not blinded to the intervention and there was no comprehensive monitoring of adverse events. Our evaluation included robust, pre-frail and frail individuals living in the community, which cannot be generalised to institutionalised older adults. While we adjusted for sex in all analyses, our study participants were mostly women, which is consistent with earlier studies showing that community exercise programmes attract mainly women [37–39]. Future studies should investigate the sex-specific effects on community-delivered pneumatic machine resistance training. Future studies could also compare resistance exercise using pneumatic machines with other exercise types (aerobic, combined etc.) and explore the longer-term effects of continued resistance training on functional performance, risk of falls, disability and mortality in older adults.

## Conclusions

Our findings extend upon earlier laboratory studies on the benefits of resistance training with pneumatic machines on muscle strength and function, that these training programmes have high adherence, can be scaled-up, and be effectively delivered by community providers at neighbourhood senior centres. Future studies could examine if including other multi-modal function-specific training to complement GT-programme can achieve better physical function in other balance, endurance and power-related tasks.

## Supplementary Information

**Additional file 1: Supplementary Figure S1.** Mean and SD of maximal isometric muscle strength outcomes from pre- to post-exercise for all participants (*n* = 234) who undertook 12 weeks of “Gym Tonic” resistance training programme. ***p < 0.001.

**Additional file 2: Supplementary Figure S2.** Mean and SD of physical function outcomes from pre- to post-exercise for all participants (*n* = 234) who undertook 12 weeks of “Gym Tonic” resistance training programme. ***p < 0.001, ns = not significant. SPPB = Short Physical Performance Battery.

## Data Availability

The datasets used and/or analysed during the current study are available from the corresponding author on reasonable request.

## Abbreviations

BMI: Body Mass Index
GS: Gait Speed
GT: Gym Tonic
PA: Physical Activity
RM: Repetition Maximum
SPPB: Short Physical Performance Battery
