# Supplementary material for: Effectiveness of a community-delivered pneumatic machine resistance training programme (Gym Tonic) for older adults at neighbourhood senior centres – a randomized controlled trial
Source: Eur Rev Aging Phys Act. 2021 Oct 7;18:21. doi: 10.1186/s11556-021-00273-x (PMC8499414; doi:10.1186/s11556-021-00273-x)
Supplement: Supplementary file 1 — Additional file 1: Supplementary Figure S1. Mean and SD of maximal isometric muscle strength outcomes from pre- to post-exercise for all participants (n = 234) who undertook 12 weeks of “Gym Tonic” resistance training programme. ***p < 0.001. [file 11556_2021_273_MOESM1_ESM.docx]

#
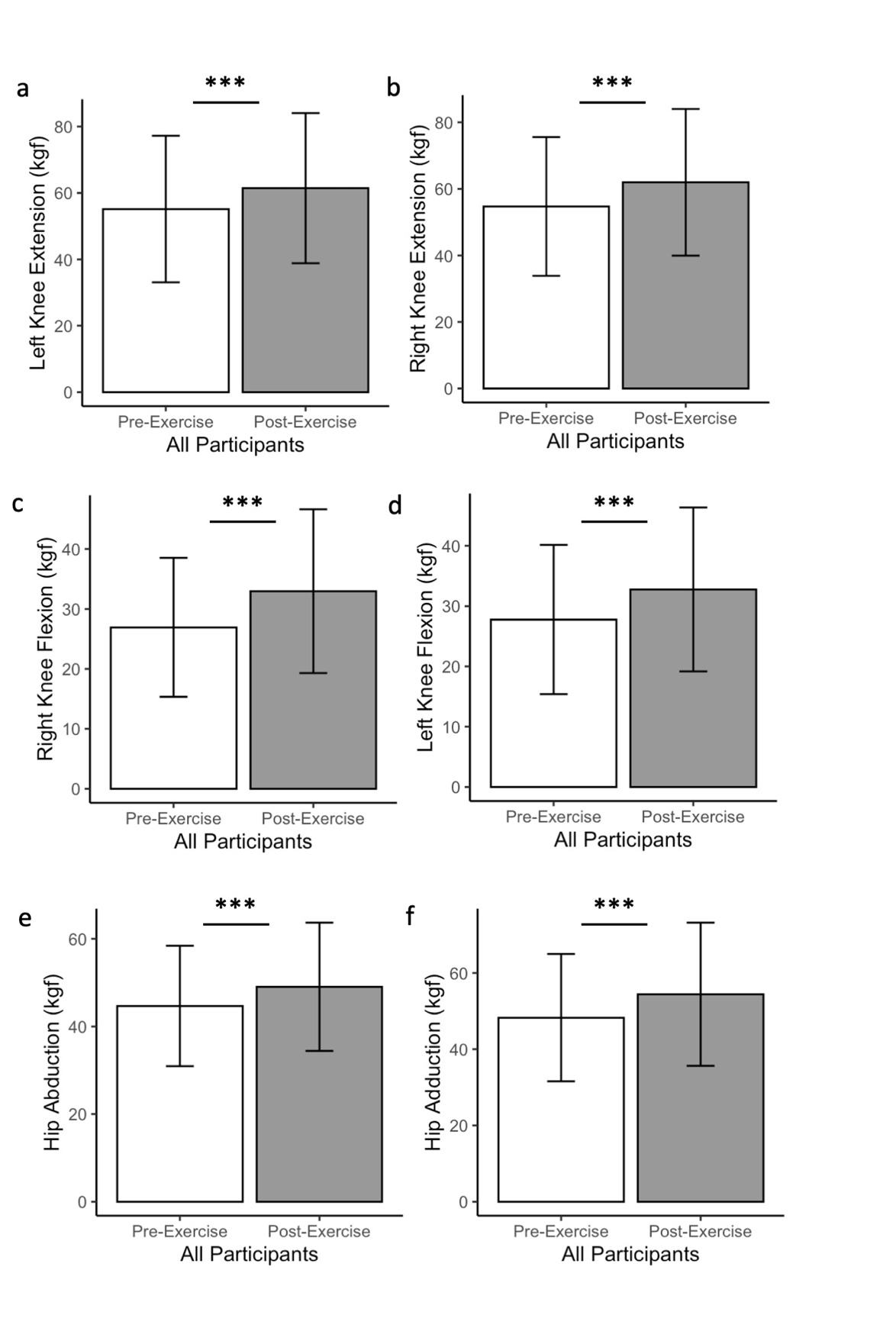
Supplementary Figures

**Supplementary Figure S1.** Mean and SD of maximal isometric muscle strength outcomes from pre- to post-exercise for all participants (*n*=234) who undertook 12 weeks of “Gym Tonic” resistance training programme. ****p*<0.001.
