# Supplementary material for: Effectiveness of a community-delivered pneumatic machine resistance training programme (Gym Tonic) for older adults at neighbourhood senior centres – a randomized controlled trial
Source: Eur Rev Aging Phys Act. 2021 Oct 7;18:21. doi: 10.1186/s11556-021-00273-x (PMC8499414; doi:10.1186/s11556-021-00273-x)
Supplement: Supplementary file 2 — Additional file 2: Supplementary Figure S2. Mean and SD of physical function outcomes from pre- to post-exercise for all participants (n = 234) who undertook 12 weeks of “Gym Tonic” resistance training programme. ***p < 0.001, ns = not significant. SPPB = Short Physical Performance Battery. [file 11556_2021_273_MOESM2_ESM.docx]

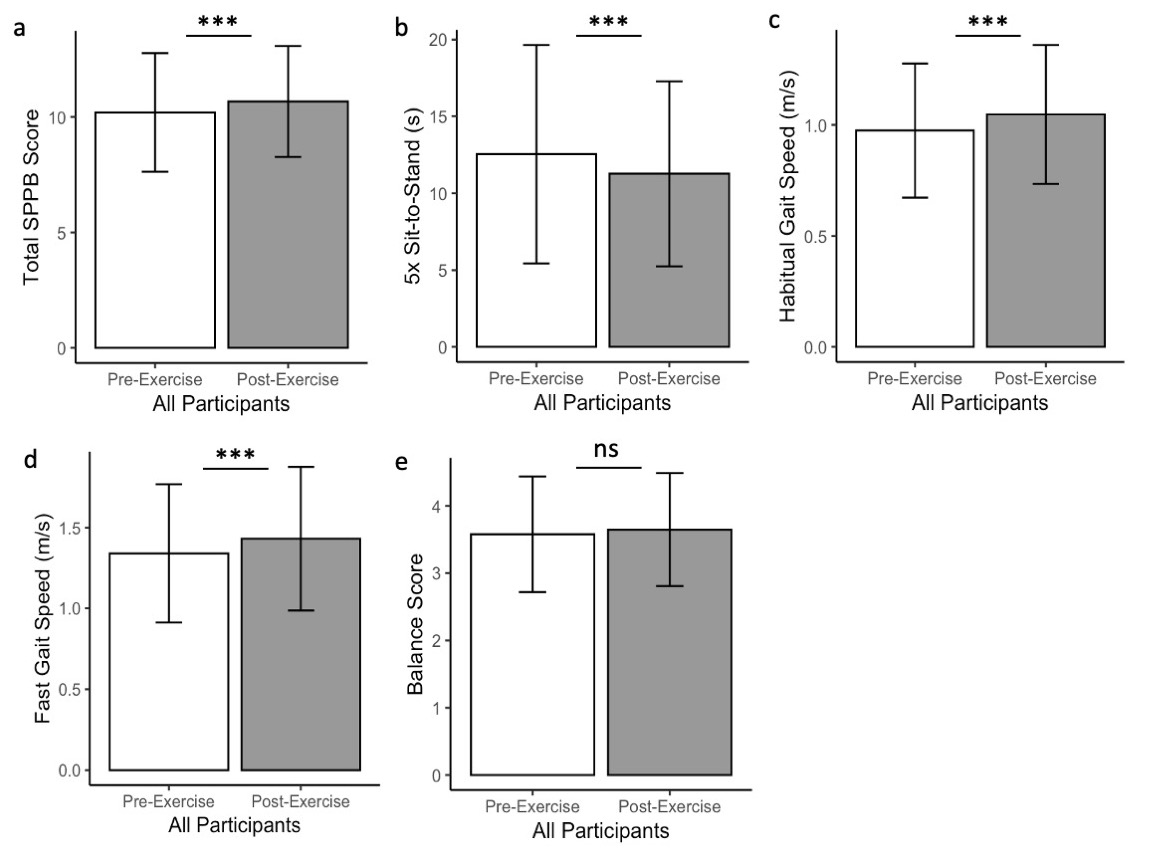


**Supplementary Figure S2.** Mean and SD of physical function outcomes from pre- to post-exercise for all participants (*n*=234) who undertook 12 weeks of “Gym Tonic” resistance training programme. ****p*<0.001, ns= not significant. SPPB = Short Physical Performance Battery.
